# Superusers experiences in using naloxone to reverse opioid overdose - A qualitative study

**DOI:** 10.1192/j.eurpsy.2022.2123

**Published:** 2022-09-01

**Authors:** K. Troberg, P. Isendahl, D. Dahlman, A. Håkansson

**Affiliations:** 1 Department of Psychiatry, Addiction Center Malmö, Malmö, Sweden; 2 Psychiatry, Department Of Clinical Sciences, Lund University, Lund, Sweden; 3 University Hospital Skåne, Department Of Infectious Disease, Malmö, Sweden; 4 Center for Primary Healthcare Research, Department Of Clinical Sciences, Malmö, Sweden

**Keywords:** Overdose management, Naloxone, Qualitative study, Opioid overdose

## Abstract

**Introduction:**

Since June 2018, multi-site overdose prevention education and naloxone distribution has been available in the County of Skåne, Sweden. Among the participants there are individuals who have used naloxone to reverse overdose on multiple occasions (three times or more). Situations of overdose management are characterized by different conditions which inevitably lead to different decisions and outcomes.

**Objectives:**

To investigate the complex interaction of individual, social, and environmental factors of opioid overdose management, how these overdose situations affect responders´ lives, what impact prior experiences may have on engagement in future overdose situations and if needs of support to deal with these situations are met.

**Methods:**

Qualitative study employing semi-structured interviews with individuals trained at needle exchange programs within the region who have used naloxone on more than two occasions to reverse opioid overdose. Interviews will be conducted during Q4 2021 and analysed thematically during Q1 2022.

**Results:**

Preliminary clinical observations point to the ambivalence between positive consequences of empowerment and pride in saving lives, and negative feelings of prior decisions and the burden to engage in future overdoses, in addition to insufficient access to support when dealing with these negative consequences.

**Conclusions:**

Not yet available.

**Disclosure:**

No significant relationships.

